# Coupling DNA Replication and Spindle Function in *Saccharomyces cerevisiae*

**DOI:** 10.3390/cells10123359

**Published:** 2021-11-30

**Authors:** Dimitris Liakopoulos

**Affiliations:** 1 CRBM, Université de Montpellier, CNRS, 1919 Route de Mende, 34293 Montpellier, France; dimitris.liakopoulos@crbm.cnrs.fr; 2 Laboratory of Biology, Faculty of Medicine, University of Ioannina, 45110 Ioannina, Greece; 3 University Research Center of loannina, University of Ioannina, 45110 Ioannina, Greece

**Keywords:** spindle, replication, S-phase checkpoint, cell cycle, yeast

## Abstract

In the yeast *Saccharomyces cerevisiae* DNA replication and spindle assembly can overlap. Therefore, signaling mechanisms modulate spindle dynamics in order to ensure correct timing of chromosome segregation relative to genome duplication, especially when replication is incomplete or the DNA becomes damaged. This review focuses on the molecular mechanisms that coordinate DNA replication and spindle dynamics, as well as on the role of spindle-dependent forces in DNA repair. Understanding the coupling between genome duplication and spindle function in yeast cells can provide important insights into similar processes operating in other eukaryotic organisms, including humans.

## 1. Introduction

Spindle formation is a hallmark of mitosis, a mitotic event par excellence. In a textbook view of the cell cycle, events occur in an orderly fashion and S-phase is completed before the G2 phase, in which cells prepare for mitosis. However, in many cases mitosis follows on directly from S-phase, especially at early embryogenesis, where cells contain large supplies of biomolecules [1]. In addition, both in budding yeast and human cells, mitosis can proceed despite the presence of unreplicated parts of the genome that are resolved after chromosome segregation [2,3].

**Figure 1:**
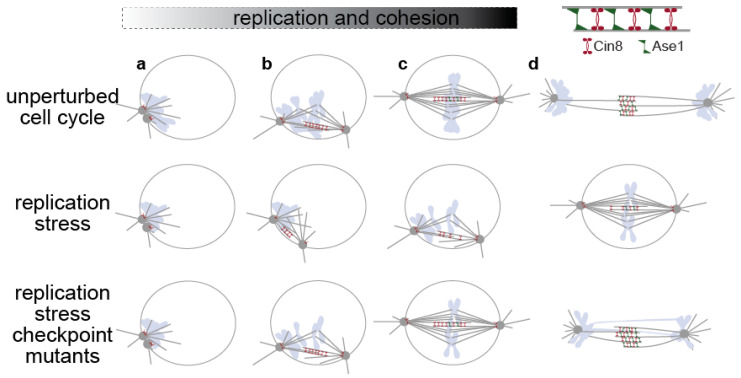
In yeast cells (only the nucleus and the spindle are depicted here), spindle formation occurs after bulk DNA replication has been completed (unperturbed cell cycle, **a**–**c**), but can occur in parallel to DNA replication upon replication stress (replication stress, **a**–**c**). The S-phase checkpoint prevents anaphase spindle elongation by inhibiting spindle-associated factors, like kinesin-5 Cin8 and Ase1/PRC1 (replication stress **a**–**c**), and by stabilizing cohesion once cohesion has been established (**c**,**d**). These mechanisms do not operate in checkpoint mutants under replication stress, and cells elongate their spindle before completion of bulk genome duplication, performing catastrophic mitosis (**d**). Nuclear membrane, SPBs and MTs in grey, chromosomes in light blue, nuclear membrane not shown at anaphase.

Upon bud emergence in budding yeast, high activity of Cdc28, the yeast Cdk1, drives the onset of DNA replication [4]. Towards the end of replication, yeast cells separate the duplicated yeast microtubule organizing centers, called Spindle Pole Bodies (SPBs) and form a mitotic spindle (Figure 1). Separation of SPBs requires Cdc28/Cdk1 and Cdc5/Polo kinase activities [5,6]. A growing array of antiparallel intranuclear microtubules (MTs) pushes the two SPBs apart, utilizing the activity of the kinesin-5 motor Cin8 and its paralogue Kip1. Later, antiparallel MTs are also stabilized by the MT-crosslinker protein Ase1, the homologue of the mammalian PRC1 protein [7]. Elongation of the mitotic spindle at anaphase occurs after completion of bulk DNA replication [3,8] and requires proteolytic deactivation of the yeast Esp1/separase inhibitor Pds1/securin by the Ubiquitin ligase APC^Cdc20^. Upon Pds1 degradation, separase cleaves Scc1/cohesin, the protein that ensures cohesion of sister chromatids, leading to anaphase chromosome segregation. Errors in spindle function, i.e., unattached chromosomes or the absence of tension at kinetochores are monitored by the Spindle Assembly Checkpoint (SAC), which prevents activation of APC^Cdc20^, Pds1 degradation and spindle elongation [9]. Finally, spindle disassembly and cytokinesis follow chromosome segregation as Cdc28 activity decreases and activation of the mitotic exit network (MEN) signals dephosphorylation of Cdc28 targets by the phosphatase Cdc14 [10].

Even the relatively simple yeast genome comprises regions that are difficult to replicate and delay the replication machinery. Examples are the tandem array of 150–200 direct repeats of ribosomal DNA (rDNA), the chromosome regions encoding tRNAs or the particular DNA structures found at telomeres [11]. More severe challenges, generally termed replication stress, may even block genome replication. These are for example obstacles that prevent progression of replication forks, ranging from cytosine methylation by alkylating agents, to DNA breaks or a lack of dNTPs induced by the drug hydroxyurea (HU).

**Figure 2:**
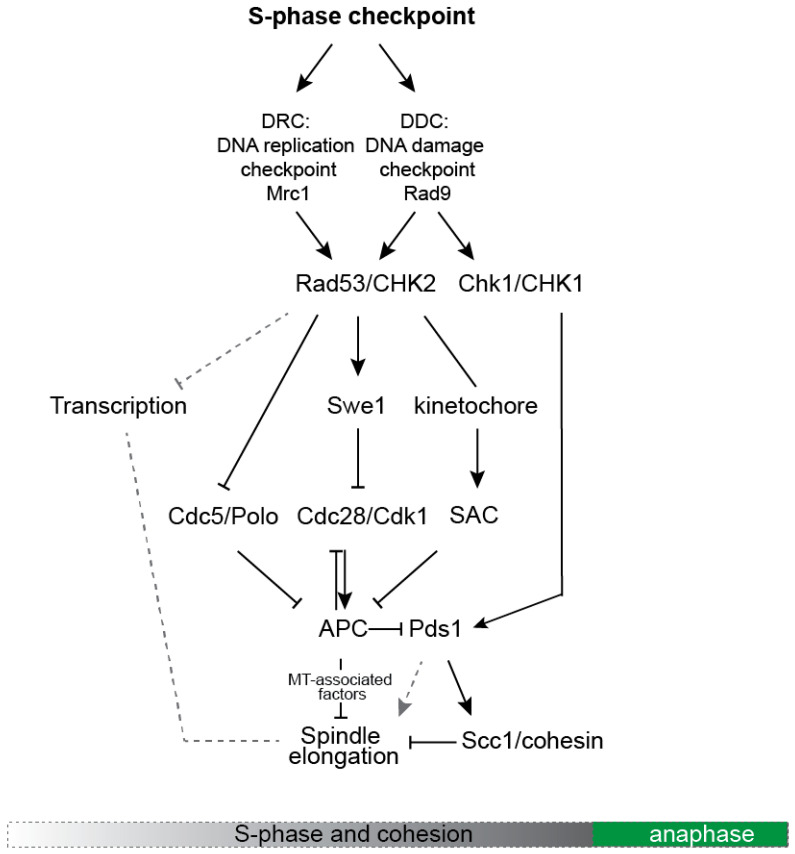
A simplified scheme depicting the main molecular pathways that control spindle dynamics in response to activation of the S-phase checkpoint.

These different types of replication stress are monitored by a checkpoint consisting of early detectors, the ATR kinase Mec1 and the ATM kinase Tel1, that transmit the alert information to the effector kinases Rad53, Chk1 and Dun1 (Figure 2) [12]. The latter are responsible for orchestrating a cellular response that includes chromosome segregation arrest, upregulation of dNTP pools, activation of DNA damage response genes, suppression of late replication origin firing and stabilization of the replication forks. Transmission of the signal from detectors to effectors passes through the mediator proteins Mrc1 or Rad9 (Figure 2). Formally, mediators define two branches of the checkpoint: Mrc1, the DNA replication checkpoint (DRC), and Rad9, the DNA damage checkpoint (DDC) branch (Figure 2) [12]. However, making this separation may be rather artificial [13,14]. Distinction between DDC and DDR will not be followed in this review and both pathways will be collectively referred to as the S phase checkpoint. Herein, the focus will be on regulation of mitotic spindle function by the S phase checkpoint.

A volume of important work over recent years has revealed that the S phase checkpoint coordinates DNA synthesis and spindle function in yeast cells. This review aims to summarize experimental findings and current understanding on main questions concerning this regulation, namely:

1. To what extent are DNA replication and spindle function coordinated in yeast cells?
2. Which molecular mechanisms control spindle dynamics upon replication failure?
3. What is the influence of spindle-dependent forces on genome stability?

## 2. Coordinating Replication with Anaphase Spindle Elongation

### 2.1. The Order of Replication and Spindle Formation in Yeast Cells

Bulk DNA replication occurs mainly before spindle assembly in normally growing yeast cells (Figure 1). Nevertheless, when DNA replication is completely absent yeast cells enter mitosis and form a mitotic spindle. For example, cells depleted of either Cdc6 or Dbf4, key components of the pre-replicative complex (pre-RC), are unable to initiate DNA replication and extend their mitotic spindle with timing similar to wild-type cells, performing catastrophic mitosis (Figure 1) [15,16]. This shows that replication is not a prerequisite for mitotic entry in yeast. In addition, replication can occur in parallel to mitosis, at least for difficult-to-replicate parts of the yeast genome. Recent evidence shows that 40% of yeast cells finish telomeric replication at early anaphase, after spindle elongation and chromosome segregation [3]; the same is likely for the replication of the rDNA locus [17]. Therefore, yeast cells allow a certain degree of flexibility regarding the timing of mitosis relative to DNA replication.

### 2.2. Cells under Replication Stress: Inhibition of Spindle Elongation

When replication is delayed or compromised, yeast cells activate the S phase checkpoint and arrest the cell cycle. Here again, activation of the S phase checkpoint does not prevent entry into mitosis but instead stops the cell cycle at either one of two specific mitotic points. The first is at pre-anaphase and responds to replication perturbations that arise prior to this stage. Here, cells arrest with a metaphase spindle. The second point is at mitotic exit, where spindle disassembly and cytokinesis are delayed until replication is complete.

Pre-anaphase arrest upon S phase checkpoint activation avoids the potentially catastrophic effects of chromosome segregation in cells with unreplicated DNA. For this, cells prevent chromosome separation by abrogating cohesin cleavage, provided that they have already duplicated a sufficiently large part of the genome for cohesion to be established [18,19]. In addition, S phase checkpoint activation attenuates spindle-dependent forces that pull chromosomes apart (Figure 1)

Evasion of cohesin cleavage relies on Chk1-dependent Pds1 phosphorylation that prevents Pds1 ubiquitylation and degradation (Figure 2) [20,21,22]. Interestingly, the S-phase checkpoint seems to control Pds1 levels also through deactivation of the Rsp5 ubiquitin ligase [23]. Moreover, absence of centromere replication and kinetochore tension leads to activation of the SAC and Pds1 stabilization as well [24,25,26]. All three mechanisms cooperate for stabilization of Pds1, showing that cohesion maintenance is important for preventing spindle elongation in cells experiencing replication stress.

Attenuation of spindle-dependent forces upon S-phase checkpoint activation becomes evident once replication becomes inhibited early in the cell cycle. Yeast cells released from G1 arrest in presence of high amounts of HU arrest with a short metaphase spindle and partly replicated chromosomes (Figure 1). Under these conditions, weakening sister chromatid cohesion (i.e., through loss of function mutations in Pds1 or Scc1) does not lead to spindle elongation [18,19,27,28,29]. In contrast, *scc1* or *pds1*∆ mutants that do not experience replication stress elongate their mitotic spindle, when blocked at pre-anaphase with fully duplicated chromosomes due to the inactivation of the APC [16,30,31]. Together, these data show that replication stress activates a mechanism that prevents spindle elongation even in absence of chromosome cohesion.

This mechanism depends on the S-phase checkpoint. When checkpoint-defective *mec1* and *rad53* mutant cells are released in HU after G1 arrest, they extend their mitotic spindle to almost normal anaphase length (3–7 µm), displaying cycles of extension, breakage, and collapse (Figure 1) [28]. In this case, spindle extension differs from normal anaphase, since it occurs in the absence of Pds1 degradation or cohesin cleavage and is independent of separase activity or APC function [18,24,32]. Here, the mitotic spindle segregates the partly replicated chromosomes randomly between daughters, leading to lethal chromosome segregation errors [28,32,33]. These data reveal that the S-phase checkpoint directly controls spindle dynamics to avoid mitotic catastrophe (Figure 1).

The S-phase checkpoint attenuates spindle function in cells with replicated chromosomes as well. This was demonstrated by studies of temperature sensitive *cdc13-1* mutant cells that display “exposed” telomeric DNA resembling double strand breaks [34]. At the restrictive temperature, *cdc13-1* cells activate the S-phase checkpoint and arrest with a mostly duplicated genome and bioriented chromosomes. Under these conditions, inactivation of cohesion leads to execution of the fast step of spindle elongation (anaphase A-chromatid separation) but not to full spindle elongation [31]. Complete chromosome separation is achieved upon deletion of the adaptor protein Rad9, suggesting that S-phase checkpoint activation prevents spindle extension, at least partly.

## 3. Mechanisms Coordinating Replication with Spindle Dynamics

### 3.1. Kinetochore Integrity Links Replication to Spindle Function

What are the molecular mechanisms underlying the pathways which prevent precocious spindle elongation? Chromosome cohesion followed by bipolar kinetochore-MT attachments and chromosome biorientation counteract the outward-directed forces of the spindle. It is therefore not surprising that kinetochore mutants as well as an aurora B kinase *ipl1* mutant bypass the S-phase checkpoint and display spindle elongation of various degrees in the presence of HU [28,35,36].

Untimely spindle extension in HU-treated *rad53* mutants seems to be a consequence of replication fork catastrophes at centromeres (*CEN*s) [37]. When such catastrophes occur, *CEN*s become susceptible to nucleases, disrupting kinetochore function and spindle force balancing mechanisms. Accordingly, mutations in the replication machinery that ensure *CEN* integrity and kinetochore formation are able to suppress spindle extension of *rad53* cells in HU. Fluorescence intensity-measurements of kinetochore components in HU-treated cells confirmed that kinetochores are correctly assembled in wild-type cells or in suppressors of the *rad53* spindle extension phenotype. In contrast, kinetochore assembly decreased with time in *rad53* cells elongating their spindle in presence of HU.

Intriguingly, while kinetochore integrity and MT-kinetochore attachments are required to prevent spindle extension, *CEN* duplication and chromosome biorientation do not appear to be essential. For example, cells with greatly delayed *CEN* duplication are still capable of arresting with short spindles after activation of the S-phase checkpoint [37]. Thus, kinetochore integrity is important to prevent spindle elongation even in cells with unduplicated *CENs*. It has been therefore proposed that monopolar attachments can resist spindle extension under early replication stress, provided MT-dependent forces are diminished [37]. This underscores the importance of attenuating spindle dynamics in order to avoid mitotic catastrophes during replication stress.

### 3.2. MT-Associated Targets of the S-Phase Checkpoint and Their Modes of Control

The molecular pathways through which the S-phase checkpoint controls spindle elongation over Cdc28, APC and Pds1 are summarized in Figure 2. Apart from Pds1, some of the first identified, spindle-associated targets of the S-phase checkpoint were Cin8 and Stu2, a member of the XMAP-215 protein family [38]. While Cin8 generates the force for spindle elongation [7], Stu2 is an essential protein required for stabilization of spindle MTs [39]. Both factors are downregulated upon activation of the S-phase checkpoint at the transcriptional and protein level (details in Figure 2). Downregulation of Cin8 and Stu2 activity rescues the spindle extension phenotype of checkpoint-defective *mec1* cells in HU while, conversely, overexpression of Cin8 results in premature spindle elongation in cells under replication stress [32,40].

The S-phase checkpoint prevents spindle elongation by downregulating the Cdc28/Cdk1 and Cdc5/Polo kinases and also by inhibiting the activity of the APC. Cdc28 downregulation is achieved through phosphorylation at Y19 by the kinase Swe1/WEE1 as well as via a Swe1-independent pathway [29,32]. Downregulation of Cdc28 activity influences also APC-dependent degradation of Cin8, Kip1 (homologs of kinesin-5) and Ase1/PRC1 [32,41,42,43]. In an unperturbed S-phase, sequential phosphorylation by Cdc28 and Cdc5 keeps the APC activator Cdh1 inactive until late anaphase, allowing accumulation of Cin8 and Kip1 and promoting spindle formation [5,44]. In the event of S-phase checkpoint activation, phosphorylation of Y19 at Cdc28 deactivates Cdc28 and alleviates Cdh1 inhibition, whereas Rad53-dependent phosphorylation of Cdc5 prevents it from inactivating Cdh1 [5,31]. Thus, the S-phase checkpoint restrains spindle elongation by keeping APC^Cdh1^ in a partially active state, limiting accumulation of Cin8, Kip1 and possibly Ase1 [31,32]. Inhibition of Cdc28 by the S-phase checkpoint prevents spindle elongation through regulation of APC^Cdc20^ as well [45], whereas reduction of Cdc28 and Cdc5 activities may also influence spindle dynamics through regulation of MT-associated factors.

As replication and cohesion establishment proceed, the forces that elongate the spindle are counterbalanced by cohesion between sister chromatids. Activation of the S-phase checkpoint at this stage prevents spindle elongation both through modulation of spindle dynamics and maintenance of cohesion. The latter occurs either directly, through phosphorylation of Pds1/securin by Chk1, or over kinetochore-dependent activation of the SAC and APC^Cdc20^ inhibition and/or inhibition of the MEN (not depicted in the figure; [46]). Preventing APC^Cdc20^ activation abrogates spindle extension via Pds1 in two ways: First, through inhibition of Esp1/separase activity and second, through a yet unclear Pds1 function in spindle stabilization that is however independent of cohesion regulation [16,47,48].

Next to posttranslational control, the S-phase checkpoint regulates spindle-associated factors at the transcriptional level as well [32,49,50]. Replication stress causes intragenic transcription of the *ASE1* gene. The result is induction of a short dominant-negative Ase1 isoform that is less stable, less abundant and stabilizes the metaphase spindle by antagonizing the full-length protein [51].

Control of the spindle dynamics late in the cell cycle (see Section 2.2) seems to depend also on Cin8 regulation by the S-phase checkpoint. Induction of double strand breaks (DSBs) in cells arrested with elongated spindles prior to mitotic exit activates a Rad9-dependent mechanism that inhibits mitotic exit and requires the SAC and components of the MEN [52,53]. This mechanism induces partial Cin8 dephosphorylation and redistribution of the kinesin from the midzone to the spindle poles (either kinetochores and/or spindle pole bodies), reversing anaphase spindle elongation [53]. These data allow the speculation that the S-phase checkpoint regulates Cin8 through phosphorylation earlier in the cell cycle as well.

Additional spindle-associated factors that are proteolytically regulated upon replication stress are the components of the yeast Aurora B chromosome passenger complex (CPC) Bir1/survivin and Sli15/INCENP [54]. In contrast to Cin8 and Ase1, activation of CPC proteolysis by the S-phase checkpoint seems to help overcome replication stress-induced arrest and promotes, rather than blocks, spindle elongation. Proteolysis of the CPC does not depend on the APC, but on a specific type of ubiquitin E3 enzymes, the SUMO-targeted ubiquitin ligases (STUbLs), that target SUMOylated proteins for degradation [55]. In mutants experiencing moderate replication stress, the SUMOylated forms of Sli15/INCENP and Bir1/survivin are ubiquitylated by the STUbL Slx5 and subsequently degraded by the proteasome [54]. SUMO-dependent degradation of Sli15 and Bir1 is thought to diminish SAC activation and help cells escape SAC-induced arrest. Indeed, SUMOylation of Bir1 and pericentromeric shugoshin Sgo1 in unperturbed cells is thought to downregulate CPC activity at kinetochores in order to allow formation of stable MT-kinetochore connections, chromosome biorientation and timely entry into anaphase [56].

## 4. Influence of Spindle-Dependent Forces on Genome Stability

Recent findings reveal that the S-phase checkpoint regulates spindle and MT dynamics not only to prevent precocious chromosome segregation but also to facilitate repair of DNA damage. The mechanisms linking DNA repair and spindle function are subject of numerous interesting recent studies but are not fully understood yet.

S-phase checkpoint signaling directly regulates kinetochore proteins [57]. The Rad53 kinase and its paralogue Dun1 phosphorylate the kinetochore protein Cep3, a DNA-binding protein that recognizes the centromeric *CDEIII* sequence as part of the CBF3 kinetochore complex. Phosphorylation of Cep3 by the S-phase checkpoint does not seem to affect binding of the protein to *CEN* sequences but relieves attachment of the *CEN* to MTs and the SPB, increasing chromatin mobility. Promoting kinesin-dependent spindle movements has been proposed to stimulate homology search during homologous recombination repair of damaged chromatin [58]. Surprisingly however, chromatin mobility induced through Cep3 phosphorylation was not required for DNA repair.

Other studies support a role of spindle-dependent chromatin mobility in the DNA repair process, but results are still difficult to reconcile. Mutations in the yeast *TUB2* gene encoding for β-tubulin give rise to DNA damage sensitivity [59,60]. This phenotype is characteristic of two specific mutations, one causing MT-destabilization (*tub2-311*) and one resulting in reduced interaction of MTs with microtubule-binding proteins (*tub2-431*, a deletion of β-tubulin C-terminal tail) [59,60]. The HU-sensitivity of these *tub2* mutants correlates with difficulty in formation of functional bipolar MT-kinetochore attachments [59]. Assuming that the latter transmit spindle-dependent forces to chromatin, these results suggest that spindle-induced chromatin mobility would be beneficial for DNA repair. However, another study showed that the *tub2-430*∆ mutation leads to increased astral MT-dependent forces on chromatin. Here, chromatin mobility impaired rather than facilitated DSB repair, suggesting that the S-phase checkpoint leads to attenuation of astral MT forces to support repair of chromosome damage [61,62].

The role of spindle-dependent forces in DNA damage was illuminated further in a study showing that cell exposure to various DNA damaging agents (MMS, zeocin and camptothecin) induces the formation of DNA damage-inducible intranuclear MT filaments (DIMs) that emanate in a monopolar fashion from SPBs [63]. These MT arrays mediate homologous recombination-type DNA repair, acting as paths for movement of damaged DNA towards nuclear pore complexes [64,65]. Importantly, kinetochore disruption by forcing transcription through the centromere induces formation of DIMs, suggesting kinetochore complexes are important for formation of these arrays. Assembly of DIMs requires the yeast α-tubulin paralog Tub3 and Kar3/kinesin-14 [58,63]. It is still unclear whether DIM assembly depends on the S-phase checkpoint (a *rad9*∆ mutant did not display significant effects in DIM formation compared to wild-type) or on S-phase checkpoint-regulated, MT-associated factors, i.e., Cin8, Ase1 or Stu2. Nonetheless, another study showed that generation of DSBs with the drug phleomycin at telophase leads to a Rad9-dependent change of the elongated telophase spindle to a dynamic, star-like distribution and a delay in exit from mitosis [53]. It is not clear, whether this star-like spindle distribution reflects formation of DIMs described above.

Finally, apart from kinetochores and astral MTs, the S-phase checkpoint modifies spindle behavior by targeting SPBs as well [66]. The effect here is mediated through the S-phase checkpoint-dependent nucleolar release of the phosphatase Cdc14 that acts on the SPB component Spc110. This protein is the intranuclear receptor of the γ-tubulin complex and a Cdc28 target. Dephosphorylation of Spc110 (and probably also other SPB proteins, i.e., Spc42) is required to bring DSBs close to the SPB for effective DSB repair. Accordingly, Spc110 variants lacking the Cdc28 phosphorylation sites display reduced capacity for DSB repair. Intriguingly however, this mechanism affected only the distance between DSB and the SPB and did not involve kinetochores. Whether Spc110 dephosphorylation by Cdc14 correlates with formation of DIMs from the SPB [63] remains to be seen.

## 5. Conclusions and Future Prospects

In yeast cells, the DNA replication and repair machineries feed back on spindle function to ensure correct timing of chromosome duplication and separation events. It is now known that this coordination requires regulation of cohesion, MT-associated factors and MT-kinetochore attachments. Still, a number of important mechanistic insights are missing. For example, the differences between wild-type spindles and spindles that elongate precociously in the absence of the S-phase checkpoint have not been studied in detail. Additional MT- and kinetochore-associated factors, apart from the few already known, seem to be phosphorylated by the S-phase checkpoint [67], with an unknown outcome. An open issue is also the force balance that prevents metaphase spindles from elongating in absence of *CEN* duplication under replication stress. Here, it is not clear to what extent semi-replicated DNA can counterbalance the force of the mitotic spindle and inhibit spindle elongation. Finally, many exciting questions are open concerning how spindle dynamics affects DNA repair. Responding to these questions will allow us to discover essential mechanisms underlying genome stability that are almost certainly conserved between yeast and cells of other eukaryotic organisms, including humans.

## Data Availability

Not applicable.
